# Expression of Ferroptosis-Related Genes Shapes Tumor Microenvironment and Pharmacological Profile in Gastric Cancer

**DOI:** 10.3389/fcell.2021.694003

**Published:** 2021-10-01

**Authors:** Shilang Xiao, Xiaoming Liu, Lingzhi Yuan, Xiao Chen, Fen Wang

**Affiliations:** ^1^Department of Gastroenterology, The Third Xiangya Hospital, Central South University, Changsha, China; ^2^Hunan Key Laboratory of Non Resolving Inflammation and Cancer, Changsha, China; ^3^Department of Gastroenterology, Human Cancer Hospital of Huaihua, Huaihua, China

**Keywords:** ferroptosis, tumor microenvironment, ferroptosis score, drug sensitivity, immunotherapy

## Abstract

**Background:** Ferroptosis is a form of regulated cell death that occurs as a consequence of lethal lipid peroxidation. A wealth of studies has demonstrated that ferroptosis profoundly modulated numerous biological behaviors of tumor. However, its natural functions in gastric cancer (GC) remain to be explored.

**Methods:** Firstly, a total of over 1,000 GC patients from the Gene Expression Omnibus (GEO) and The Cancer Genome Atlas (TCGA) database were included in our study. Secondly, 32 ferroptosis-related genes were extracted from the ferrDb website. Then, unsupervised clustering was performed to classify patients into three distinct ferroptosis-related clusters. Subsequently, we systematically and comprehensively explored the biological characteristics of each cluster. Finally, we constructed a scoring system, named ferroptosis score, to quantify each cluster and also investigated the predictive therapeutic value of the ferroptosis score for chemotherapy and immunotherapy.

**Results:** Based on the expressions of 32 ferroptosis-related genes, three distinct ferroptosis-related subtypes with various biological characteristics were determined. Integrated analysis showed that cluster 1 is a microsatellite instability (MSI)-like subtype, cluster 2 is an epithelial–mesenchymal transition (EMT)-like subtype, while cluster 3 tends to be a metabolic-like subtype. Prognostic analysis revealed that patients in cluster 2 had a worse overall survival and relapse-free survival. The distribution of the ferroptosis score was significantly different in clusters and gene clusters. The ferroptosis score could predict the biological characteristics of each cluster, the stromal activity, and progression of tumor. The low ferroptosis score group was characterized by the activation of antigen processing and presentation, DNA damage repair pathways, and metabolic pathways, while the high ferroptosis score group was characterized by stromal activation. In response to anticancer drugs, the ferroptosis score was highly negatively associated with drugs targeting MAPK signaling and PI3K/mTOR signaling, while it was positively correlated with drugs targeting the cell cycle, mitosis, and metabolism. Finally, we also proved that the ferroptosis score could serve as a reliable biomarker to predict response to immunotherapy.

**Conclusion:** This work revealed that tumor cells and their surrounding microenvironment could be shaped by varying the activation degrees of ferroptosis. Establishing ferroptosis-related subtypes would guide in predicting the biological features of individual tumors and selecting appropriate treatment protocols for patients.

## Introduction

Gastric cancer (GC) is the fifth most diagnosed malignancy worldwide with over 1 million new cases annually. Unfortunately, due to the difficulty of an early diagnosis, the mortality rate is quite high, causing 78,400 deaths in 2018 (Smyth et al., 2020). Gastric cancer is a heterogeneous disease with numerous genetic mutations and epigenetic alterations, breaking the balance between the oncogenic and tumor suppressor pathways. Recent research has shown that the imbalance between the proliferation and death of cancer cells is implicated in the occurrence and progression of GC.

Ferroptosis is a process of regulated cell death attributed to lethal lipid peroxidation (Stockwell et al., 2017). Compared with other cell death modes such apoptosis, necrosis, and autophagy, ferroptosis shows distinct characteristics in terms of morphology, biochemistry, and genetics. Typical manifestations include the rupture and blistering of cell membranes, normal-sized nuclei without condensed chromatin, and peculiar mitochondrial changes, such as shrinkage, increased membrane density, decreased or disappearance of mitochondrial ridges, and rupture of the outer mitochondrial membranes (Dixon et al., 2012). The initiation and execution of ferroptosis lie at the intersection of amino acid, lipid, and iron metabolism. Cystine is transported through the membrane by system XC^–^ to synthesize glutathione (GSH), which is a necessary cofactor of *GPX4* for eliminating lipid peroxides. Depletion of GSH or cystine and GPX4 knockout would cause the accumulation of reactive oxygen species (ROS), resulting in lipid peroxidation and triggering ferroptosis (Angeli et al., 2017). In lipid metabolism, polyunsaturated fatty acids (PUFAs), which contain bis-allylic hydrogen atoms that can be readily abstracted, are esterified into membrane phospholipids and undergo oxidation to destruct the lipid bilayer and affect membrane function (Chen X. et al., 2021). Besides, iron is responsible for the accumulation of lipid peroxides through at least two mechanisms: the production of ROS through the iron-dependent Fenton reaction and the activation of iron enzymes, such as lipoxygenase. Therefore, iron metabolism is also involved in the execution of ferroptosis (Yang et al., 2016). In addition, sensitivity to ferroptosis is also regulated by other biological processes, such as the biosynthesis of nicotinamide adenine dinucleotide phosphate (NADPH) and coenzyme Q10 (Shimada et al., 2016a, b; Viswanathan et al., 2017). Accumulating evidence has demonstrated that ferroptosis not only acts as a novel form of cell death but also regulates numerous biological behaviors of tumors, including epithelial–mesenchymal transition (EMT), immune response, genomic mutation, progression, and drug resistance (Granofszky et al., 2018; Chen et al., 2020; Yan et al., 2021).

Although a large number of studies have focused on genetic mutations and epigenetic alterations of tumor cells, a wealth of novel research has demonstrated that the microenvironment, on which tumor cells depend for subsistence and growth, plays an indispensable role in the biological behaviors of tumors (Quail and Joyce, 2013). The tumor microenvironment (TME) is a complex infrastructure composed of numerous cells and factors including infiltrating inflammatory cells, bone marrow-derived hematopoietic and endothelial progenitor cells, carcinoma-associated fibroblasts, extracellular matrix, and secreted factors like cytokines, lipid mediators, and growth factors (Pitt et al., 2016). Communication between the TME and cancer cells is through two major ways: contact-independent mechanisms *via* soluble molecules and contact-dependent mechanisms. Such communication could induce biological changes, such as angiogenesis and immune evasion, which promote proliferation and favor the survival of tumor cells (Pitt et al., 2016). Furthermore, increasing evidence has revealed the characteristics of the TME, such as immune cell infiltration, and also found that the activation degrees of the stroma could influence the response to immunotherapy (Pitt et al., 2016; Tauriello et al., 2018). Therefore, identifying distinct TME phenotypes or biomarkers reflecting the characteristics of different phenotypes would be of significance to predict the response to immunotherapy of individual patients.

The release of damage-associated molecular patterns (DAMPs) during cell death regulates antitumor immunity, implying that the immunity landscape could be shaped by ferroptosis activity, to some extent (Sun et al., 2020). In recent studies, ferroptosis seemed to play a dual role in antitumor immunity. Multiple DAMPs, such as HMGB1, KRAS-G12D, and damage-induced DNA released by ferroptotic cells led to the polarization of macrophages to the M2 phenotype and the stimulation of tumor growth (Wen et al., 2019; Dai et al., 2020a, b). Besides, ferroptosis promoted by T cell-expressed CD36 reduced the release of T cell-generated cytotoxic cytokines, weakening the antitumor effect of T cells, particularly when combined with anti-PD-1 (Ma et al., 2021). Moreover, activation of 12/15-lipoxygenase limited the maturation of dendritic cells and dampened the differentiation of Th17 cells (Rothe et al., 2015). However, some studies also demonstrated that ferroptosis facilitated antitumor immunity. In a pan-cancer analysis, tumors with elevated sensitivity to ferroptosis exhibited higher enrichment scores of CD8^+^ T cells (Tang et al., 2020).

On the ferrDb website, ferroptosis-related genes could be classified into three categories: (1) drivers, promoting ferroptosis; (2) suppressors, inhibiting ferroptosis; and (3) markers, regulating ferroptosis (Zhou and Bao, 2020). These genes control the activity of ferroptosis and mediate the downstream biological behaviors. Since ferroptosis is a complicated biological process and its biological effects could not be depicted clearly by a single regulator, comprehensive biological changes mediated by multiple ferroptosis regulators would allow us to better understand the functional role of ferroptosis in tumor cells and the TME. In this study, we integrated the genomic information of over 1,000 GC patients to identify three ferroptosis status and describe the biological characteristics of each. Then, the correlation between the ferroptosis-related phenotypes and consensus molecular subtypes of GC was examined. We found that the TME could be shaped by different ferroptosis activities into three subtypes: EMT-like subtype, microsatellite instability (MSI)-like subtype, and metabolic-like subtype. Each subtype has distinct enriched pathways and TME characteristics. To characterize and quantify each subtype, we established a scoring system named ferroptosis score. Finally, we demonstrated the predictive value of the ferroptosis score for the efficacy of chemotherapy and immunotherapy.

## Materials and Methods

### Data Source and Pre-processing

All public gene expression data and corresponding clinical characteristics were extracted from the Gene Expression Omnibus (GEO) and The Cancer Genome Atlas (TCGA) database. A total of five GC cohorts, namely, GSE14549, GSE34942, GSE57303, GSE62254, and TCGA-STAD, were enrolled in this study. Since all microarray data gathered in our study were from the Affymetrix platform, we downloaded the raw ‘‘CEL’’ file of each cohort and adopted the multi-array averaging method for background adjustment and quantile normalization. RNA sequencing data in the raw count format were obtained from the UCSC Xena Browser^1^. Then, the raw count value was normalized using the Deseq2 package for further analysis. We used the “ComBat” algorithm to remove batch effects in the microarray data (Leek et al., 2012). Both somatic mutation data and copy number alteration data were obtained from TCGA database.

### Identification of Ferroptosis-Related Genes

Ferroptosis-related genes were downloaded from the FerrDb database that provides comprehensive and up-to-date knowledge for ferroptosis regulatory markers and related diseases. A total of 259 ferroptosis-related genes were obtained. Besides, in the pre-filter stage, the ferroptosis-related genes exhibiting low median absolute deviation (MAD < 1) in the merged GEO cohort were excluded. Finally, 32 ferroptosis-related genes were selected for further analysis.

### Unsupervised Clustering for 32 Ferroptosis-Related Genes

Based on the expressions of 32 ferroptosis-related genes, consensus clustering was performed using the “ConsensusClusterPlus” package to classify GC patients into three subgroups (Wilkerson and Hayes, 2010). The selected clustering algorithm was *K*-means and the distance was measured by Euclidean metric. Besides, we also performed the above steps 1,000 times to guarantee the stability of classification. Next, we applied another unsupervised algorithm, the non-negative matrix factorization (NMF), to validate the clustering efficacy of consensus clustering.

### GSVA and Functional Annotation

To figure out the biological characteristics of each ferroptosis status, we used the “GSVA” package for gene set variation analysis (GSVA). The gene sets of “c2.cp.kegg.v7.2.symbols” were downloaded from the MSigDB database (Hänzelmann et al., 2013). The “clusterProfiler” package was performed to conduct functional annotation for differentially expressed genes (DEGs) (Yu et al., 2012).

### Estimation of Immune Cell Infiltration

A single-sample gene set enrichment analysis (ssGSEA) algorithm was introduced to quantify the relative infiltration of 28 immune cell types within the TME. The featured gene panels for each distinct immune cell were obtained from the study of Charoentong et al. (2017). The enrichment scores calculated using ssGSEA represented the abundance of immune cells within the TME in each sample.

Establishment of Ferroptosis Scores to Evaluate Individual GC Patients

1) Differential expression analysis was performed using the “Limma” R package to identify the ferroptosis-related DEGs among three distinct subtypes (Ritchie et al., 2015). An adjusted *p* < 0.001 was considered as the significance criterion for determining DEGs.
2) Univariate Cox regression analysis was performed to calculate the risk ratio for each ferroptosis-related DEG. DEGs with significant prognostic values were selected for further analysis.
3) After obtaining the prognostic value of each DEG, we applied an algorithm similar to the Gene Expression Index (GGI) to calculate the ferroptosis score: ferroptosis score = (beta_*i*_ × Exp_*i*_), where “i” is the ferroptosis subtype-related gene (Sotiriou et al., 2006; Chen H. et al., 2021).

### Association Between Ferroptosis-Related Phenotypes or Ferroptosis Score With Other Biological Processes

The featured gene panels associated with tumor-related biological processes were obtained from the study of Mariathasan et al. (2018) which include: (1) immune checkpoint; (2) EMT markers, including EMT1, EMT2, and EMT3; (3) angiogenesis signature; (4) pan-fibroblast transforming growth factor beta (TGF-β) response signature (Pan-F-TBRS); (5) DNA damage repair; (6) mismatch repair; (7) nucleotide excision repair; (8) DNA replication; and (9) antigen processing and presentation (Şenbabaoğlu et al., 2016). Besides, the Asian Cancer Research Group (ACRG) constructed a panel of gene signatures representing the biological characteristics of GC molecular subtypes, which include: (1) EMT positive signature; (2) EMT negative signature; (3) MSI signature; (4) proliferation signature; (5) cytokine signature; (6) gastric tissue signature; and (7) p53 activity signature (Cristescu et al., 2015). The enrichment scores of the above gene signatures were compared among the different ferroptosis-related phenotypes, and an association analysis between the ferroptosis score and the tumor-related process was also performed.

### Collection of Datasets With Immunotherapy

Due to the large sample size and comprehensive clinical information, the expression data in raw count format and detailed clinical information of the IMvigor210 cohort were downloaded from http://research-pub.Gene.com/imvigor210corebiologies (Mariathasan et al., 2018). The raw count data were normalized using the “Deseq2” R package for further analysis.

### Association Between Ferroptosis Score and Drug Sensitivity

The transcriptional expression data of over 1,000 cancer cell lines, the drug response of each cell line to distinct drugs measured by IC_50_, and the pathways targeted by each drug were downloaded from the Genomics of Drug Sensitivity in Cancer database (GDSC)^2^ (Yang et al., 2013). Correlation between the ferroptosis score and drug response activity was calculated using Spearman’s correlation analysis. We set | Rs| > 0.15 and *p* < 0.05 as significant correlation.

### Statistical Analysis

Spearman’s and distance correlations were used to calculate the correlation coefficients between each ferroptosis-related gene and between each type of infiltrating immune cell and each ferroptosis-related gene. Wilcoxon’s test or the Kruskal–Wallis test was used for comparison of differences between two groups or among three groups, respectively. Based on the association between the ferroptosis score and patient’s survival, the cutoff point of the survival information for each dataset was determined using the survminer R package. The ferroptosis score was dichotomized by the “surv-cutpoint” function of the survminer package, and all potential cutoff points were repeatedly tested in order to find the maximum rank statistic. Under the selected maximum log-rank statistics, patients were classified into the high ferroptosis score group and the low ferroptosis score group to reduce the calculated batch effect. The Kaplan–Meier method and log-rank tests were utilized to compare the survival differences between grouped patients. A univariate Cox model was used to calculate the risk ratio of each ferroptosis-related gene and the ferroptosis-related DEGs. A multivariate Cox model was performed to assess whether the ferroptosis score could serve as a robust and independent predictive biomarker. A receiver operating characteristic (ROC) curve was generated and the area under the curve (AUC) was calculated with the “survival ROC” R package to assess the specificity and sensitivity of the ferroptosis scores. The R package “maftool” was used to depict the mutation landscape of the ferroptosis-related genes. We considered a two-sided *p* < 0.05 as statistically significant. The above statistical analysis and visualization were achieved with R 4.0.1 software.

## Results

### Ferroptosis-Related Genes Were Involved in the Development of Gastric Cancer

A total of 32 ferroptosis-related genes, including 7 drivers, 11 suppressors, and 14 markers, were screened using MAD > 1 as the threshold, as described in section “Materials and methods.” Among the 32 ferroptosis-related genes, *CDKN2A*, *GDF15*, *TRIB3*, *MYB*, *NOX4*, *PSAT1*, *SCD*, *SCL7A11*, *CXCL2*, and *NNMT* were significantly upregulated in tumor samples, while *AKR1C1*, *AKR1C2*, *MT1G*, *ATF3*, *SLC2A12*, *AKR1C3*, *CAV1*, *VLDLR*, *RGS4*, *DPP4*, *BNIP3*, *TFAP2C*, *CA9*, *PROM2*, and *ENPP2* were significantly upregulated in normal tissues (Supplementary Figure 1A). Principal component analysis revealed that tumor tissues and normal tissues could be well-separated by the expressions of these genes (Supplementary Figure 1B), indicating the important role of these implied ferroptosis-related genes in the occurrence of GC. Furthermore, the expressions of ferroptosis-related genes in different grades of GC were also explored. The results showed that nine genes were upregulated and three genes were downregulated in advanced GC (stages III and IV) (Supplementary Figure 1C). Univariate Cox regression analysis revealed that 12 out of 32 ferroptosis-related genes were directly associated with overall survival of GC patients (Supplementary Figure 1D). The above results also demonstrated that ferroptosis-related genes were involved in the progression of GC.

### Landscape of Genetic Alteration of Ferroptosis-Related Genes in Gastric Cancer

We examined the incidence of somatic copy number variations (CNVs) and somatic mutations of these genes. Among 437 samples, 25.63% of patients carried mutations of ferroptosis-related genes. *DUOX2* showed the highest mutation frequency, followed by *CDKN2A*, *ZEB1*, *ENPP2*, and *SLC2A3*, while *ATF3*, *CXCL2*, *MT1G*, *SCD*, *AKR1C2*, *IL33*, and *IL6* did not show mutations (Supplementary Figure 2A). In addition, we found no significant copy number alterations for almost all genes, implying that CNVs may not be a major cause of the differential expressions of these genes between normal tissues and tumor tissues and between individual tumors (Supplementary Figure 2B). Heterogeneous expressions of ferroptosis-related genes may result from other factors, such as DNA methylation and transcriptional factors (Luxen et al., 2008; Jiang et al., 2017; Kang et al., 2020). After pairwise correlation of the expressions of ferroptosis-related genes (Supplementary Figure 1E), we discovered that *DUOX2*, a driver of ferroptosis, was positively associated with several suppressors, including *AKR1C3*, *SLC7A11*, and *PROM2*, other than multi-drivers, such as *DPP4*, *ATF3*, and *CDKN2A*. Besides, the expressions of *CAV1*, *ZEB1*, *RGS4*, *NNMT*, *NOX4*, *BNIP3*, and *ENPP2* correlated with each other, and some of which have been demonstrated (Kanska et al., 2017). These findings imply a potential regulatory relationship among these genes, and the cross-talk may play a role in generating different phenotypes in individuals with tumors.

### The Biological Characteristics of Each Ferroptosis-Related Phenotype

**FIGURE 1:**
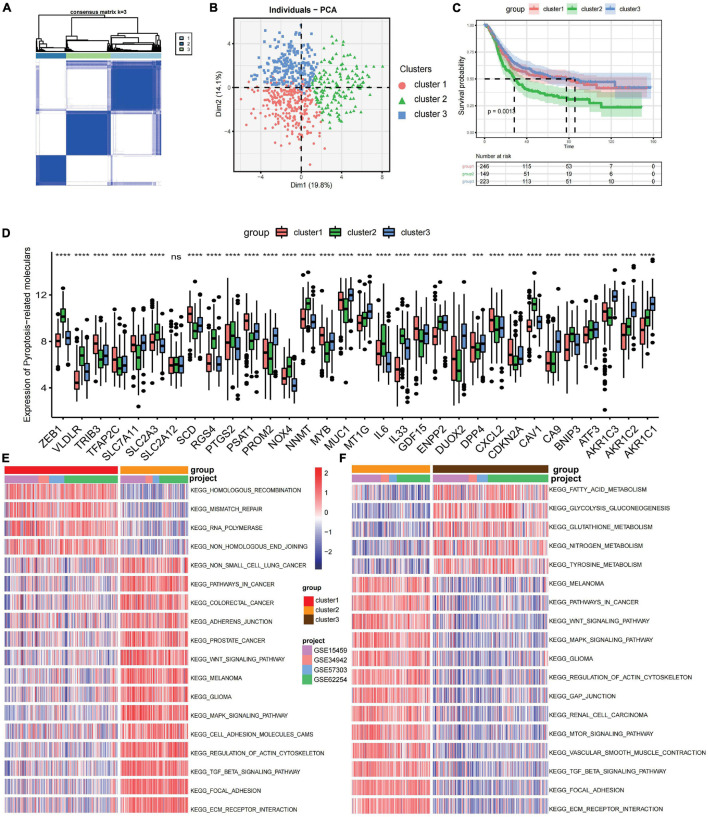
Prognosis and biological characteristics of each ferroptosis-related subtype. **(A)** Consensus clustering matrix for *k* = 2. **(B)** Principal component analysis for the transcriptome profiles of three ferroptosis-related subtypes. **(C)** Kaplan–Meier analysis of patients in the three different ferroptosis-related subtypes. **(D)** Expression profiles of 32 ferroptosis-related genes in three subtypes. *ns*, no significance. ***p < 0.001; ****p < 0.0001, as determined by the Kruskal–Wallis test. **(E,F)** Enrichment degree of biological pathways in the different ferroptosis-related subtypes shown in a heatmap: cluster 1 vs. cluster 2 **(E)** and cluster 2 vs. cluster 3 **(F)**.

The R package of ConsensusClusterPlus was used to classify patients in merged GEO cohorts into three subgroups based on the expressions of 32 ferroptosis-related genes. Three distinct clusters were eventually identified using the unsupervised clustering method: 252 cases in cluster 1, 151 cases in cluster 2, and 223 cases in cluster 3 (Figure 1A). Principal component analysis showed the existence of distinct transcriptome profiles among the three clusters (Figure 1B). The expression patterns of these ferroptosis-related genes are shown in Figure 1D. Cluster1 was characterized by increased expressions of *SLC2A12*, *CDKN2A*, *DPP4*, *TFAP2C*, *SCD*, *TRIB3*, *MYB*, *GDF15*, *SLC7A11*, and *PSAT1*. Cluster 2 displayed high expressions of *NOX4*, *NNMT*, *IL33*, *BNIP3*, *VLDLR*, *RGS4*, *CAV1*, and *ZEB1*. Cluster 3 exhibited significant increases in the expressions of *DUOX2*, *AKR1C1*, *AKR1C2*, *AKR1C3*, *MT1G*, *MUC1*, *CA9*, and *PROM2* (Figure 1D). Prognostic analysis of the three clusters showed that patients in cluster 2 have a shorter survival compared to those in the other clusters (Figure 1C).

**FIGURE 2:**
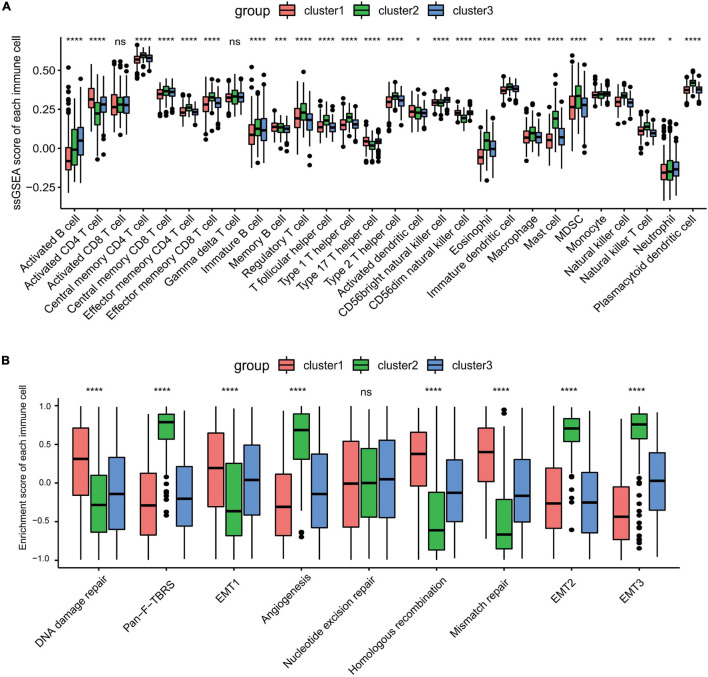
Tumor microenvironment immune cell infiltration and transcriptome traits in distinct ferroptosis-related subtypes. **(A)** The abundance of each infiltrating immune cell in three distinct ferroptosis-related subtypes. *ns*, no significance. *p < 0.05; ***p < 0.001; ****p < 0.0001, as determined by the Kruskal–Wallis test. **(B)** Different activation status of the stromal activation pathways in three clusters, with *asterisks* representing the statistical *p*-values (****p < 0.0001). *ns*, no significance.

Gene set variation analysis (GSVA) was performed to explore the biological behaviors of the three clusters. As shown in Figures 1E,F, carcinogenic pathways and stromal activation pathways, such as the Wnt signaling pathway, mitogen-activated protein kinase (MAPK) signaling pathway, TGF-β signaling pathway, and focal adhesion pathway, were significantly enriched in cluster 2. Cluster 1 showed enriched pathways related to the replication of DNA, such as the homologous recombination pathway, mismatch repair pathway, and non-homologous end joining pathway, while energy metabolism pathways related to glucose, fatty acid, and amino acid were enriched in cluster 3. In the subsequent analysis of infiltrating lymphocytes within the TME using the ssGSEA algorithm (Figure 2A), although cluster 2 showed worse survival outcomes, immune cell infiltration, such as central memory CD4 T cell, Th1 cell, natural kill cell, and plasmacytoid dendritic cell, was significantly enriched. Previous studies have demonstrated that tumors with an immune-excluded phenotype could also exhibit a high infiltration of immune cells, but these immune cells were trapped in the stroma surrounding tumor cell nests rather than penetrating into the parenchyma (Chen and Mellman, 2017; Zhang et al., 2020). The GSVA revealed that the stromal activation pathways were significantly enriched in cluster 2 compared to the other clusters. Subsequent analysis further demonstrated that signaling reflecting stromal activation, such as the EMT, TGF-β, and angiogenesis pathways, were also activated in cluster 2 (Figure 2B). Therefore, we speculated that the function of infiltrating lymphocytes was suppressed in cluster 2 due to the activation of the stroma, resulting in an inferior prognostic outcome. Both clusters 1 and 3 were characterized by decreased infiltration of regulatory T cells and myeloid-derived suppressor cells (MDSCs), which had a negative impact on immunocompetence (Fan et al., 2020), while the abundance of activated CD4 T cells and activated B cells increased in clusters 1 and 3, respectively. These results suggested that cluster 2 is immunosuppressive while the other clusters are relatively immune-activated and that clustering based on ferroptosis-related genes is closely correlated with prognosis and the immune microenvironment in GC.

### Association Between Consensus Molecular Subtypes and Ferroptosis-Related Subtypes

**FIGURE 3:**
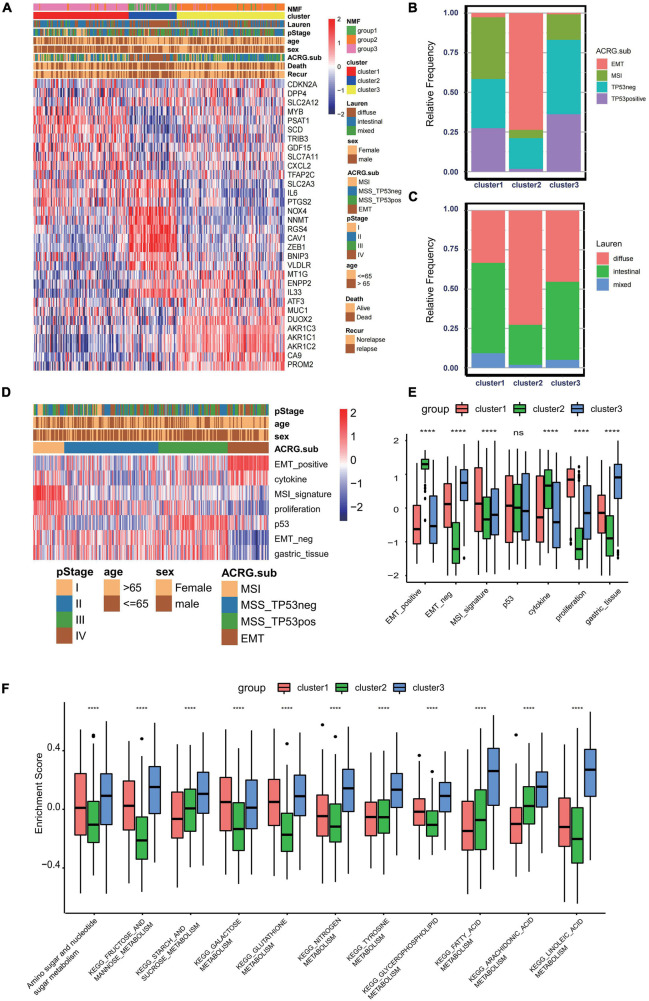
Association between consensus gastric cancer molecular subtypes and the ferroptosis-related clusters. **(A)** Expression profiles of the 32 ferroptosis-related genes in the Asian Cancer Research Group (ACRG) cohort. The ferroptosis-related clusters, ACRG molecular subtypes, histology subtype, tumor stage, survival status, relapse status, gender, and age were used as patient annotations. **(B)** Relative frequency of the ACRG molecular subtypes in the three clusters. **(C)** Relative frequency of Lauren’s histological subtypes in three clusters. **(D)** Biological characteristics of each ACRG molecular subtype shown in heatmaps. **(E)** Different activation status of the gene signatures characterized by distinct ACRG molecular subtypes in ferroptosis clusters. ****p < 0.0001). *ns*, no significance. **(F)** Different enrichment status of numerous metabolism pathways in the Kyoto Encyclopedia of Genes and Genomes (KEGG) database in three distinct ferroptosis clusters (****p < 0.0001). *ns*, no significance.

To further explore the clinical traits and biological behaviors in the three clusters, we paid more attention to the ACRG cohort, which provided a large sample size with more comprehensive clinical information. Similar to the merged GEO cohorts, unsupervised clustering also discovered three distinct subgroups within the ACRG cohort based on the expressions of 32 ferroptosis-related genes (Supplementary Figures 3A–D). In the ACRG cohort, the expression patterns of the ferroptosis-related genes in the three subtypes are shown as heatmaps and were proven to be similar to those of the combined GC cohort (Figure 3A). Besides, we also discovered that the EMT subtypes were remarkably associated with cluster 2, while the MSI subtype was more relevant to cluster 1 (Figure 3B). In addition, patients in cluster 2 were characterized by the diffuse subtype exhibiting poor differentiation, while others tended to be of intestinal subtype with better differentiation (Figure 3C). A recent study has revealed that patients with a diffuse histological subtype had worse overall survival, while the MSI subtype is usually associated with better clinical outcomes. Therefore, tumors in cluster 2 were characterized by stromal activation, high malignancy, and rapid progression. Moreover, prognostic analysis also showed that cluster 2 was markedly linked with a poorer overall survival and a worse relapse-free survival (Supplementary Figures 3E,F). The histogram of the frequency distribution revealed that almost all EMT subtypes were in cluster 2, while most MSI subtypes were in cluster 1 (Figure 3B). This also validated that cluster 2 is closely related to the EMT subtype while cluster 1 is more associated with the MSI subtype.

GC can be divided into four consensus molecular subtypes (ACRG subtype): (1) MSI subtype with hypermutation status, elevated expression of the MSI signature, cytokine signaling, and cell proliferation signaling; (2) EMT subtype with activation of the EMT signature and abatement of proliferation signatures; (3) MSS/TP53- (TP53-inactive type) with functional loss of TP53; and (4) MSS/TP53+ (TP53-active type) with full TP53 activity (Figure 3D; Cristescu et al., 2015). In the merged GC cohort, GSVA was performed to calculate the activation degree of the aforementioned signatures in the three clusters. The enrichment scores of the EMT-related signatures were significantly elevated in cluster 2, while those of the MSI-related signatures, including the MSI and proliferation signatures, were significantly higher in cluster 1. In addition, MSI was always concurrent with the overexpression of proteins involved in DNA damage repair. Enrichment analysis also indicated that the pathways involved in DNA damage repair, such as homologous recombination, base excision repair, and non-homologous end joining pathways, were significantly enriched in cluster 1 (Figure 3E). A distinct metabolic molecular subtype of GC with evident metabolic dysregulation was proposed in the study of Lei et al. (2013), in which enrichment of multiple metabolism signatures was pronounced. Interestingly, activation of the metabolism-related pathways was one of the major characteristics of cluster 3 (Figure 3F). Metabolism signatures, such as fatty acid metabolism, glutathione metabolism, and linoleic acid metabolism, were significantly activated in cluster 3 compared to that in other clusters. These results further demonstrated that cluster 1 is a MSI-like subtype, cluster 2 is an EMT-like subtype, while cluster 3 is more likely to be a metabolic-like subtype.

**FIGURE 4:**
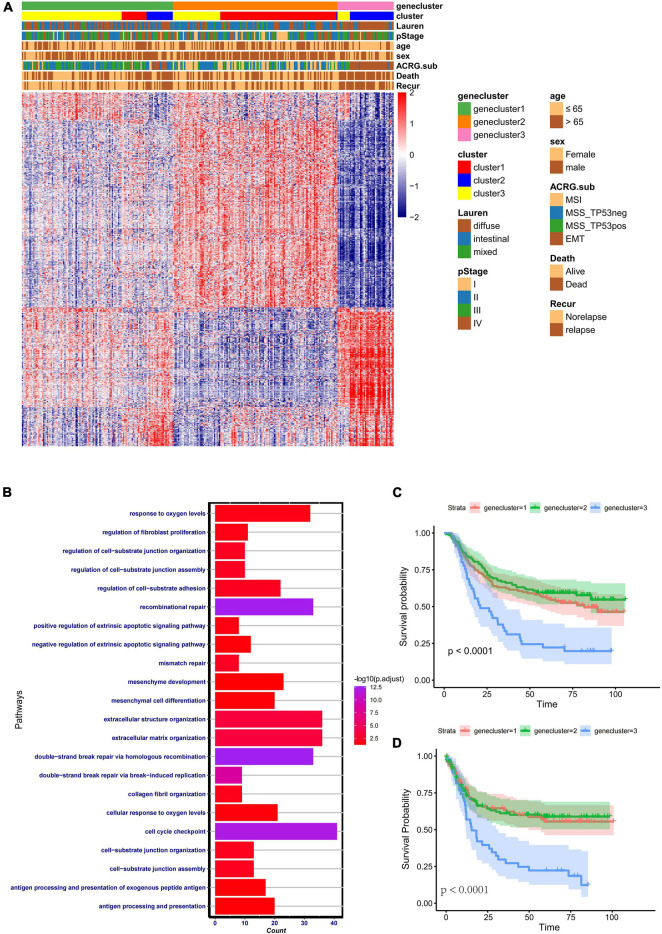
Clinical features and transcriptome characteristics of distinct gene clusters. **(A)** Unsupervised clustering for the 933 overlapping ferroptosis-related genes classified patients in the Asian Cancer Research Group (ACRG) cohort into three gene clusters with distinct biological characteristics. The gene clusters, ferroptosis-related clusters, ACRG molecular subtypes, histology subtype, tumor stage, survival status, relapse status, gender, and age were used as patient annotations. **(B)** Gene Ontology (GO) biological pathway enrichment analysis for the 933 overlapping phenotype-related genes. **(C)** Kaplan–Meier analysis of patients in three different ferroptosis-related gene clusters in the ACRG cohort. **(D)** Relapse-free survival analysis of the patients in three different ferroptosis-related gene clusters in the ACRG cohort.

To further identify the functional roles of the three patterns identified above, 933 phenotype-related DEGs were determined using the “limma” package. Gene Ontology (GO) enrichment analysis of the DEGs was performed with the “ClusterProfiler” package (Figure 4B). The results of the enrichment analysis indicated that the DEGs showed enrichment in biological processes related to DNA damage repair, stromal-related process, cellular response to oxygen, and antigen process and presentation. To further validate this differential regulation, unsupervised clustering analysis based on these 933 phenotype-related genes was performed in the ACRG cohort. Three genomic subtypes, termed geneclusters 1–3, were identified (Figure 4A). We observed that tumors in genecluster3 were related to poor differentiation and enriched in diffuse histological subtypes, while the other subtypes were enriched in genecluster 1 and genecluster 2. Besides, patients with dead status and EMT molecular subtypes were more likely clustered into genecluster 3, while patients with MSI subtypes were mostly classified into genecluster 2. Heatmaps also indicated that each gene cluster had its own gene signatures. The clinical traits and genomic characteristics of geneclusters 1–3 were consistent with those in cluster 1–3, revealing the existence of three distinct phenotypes clustered based on ferroptosis-related genes. In addition, patients in genecluster 3 (45 patients) had worse overall survival and relapse-free survival, similar to those in cluster 2 (Figures 4C,D).

### Construction of Ferroptosis Gene Signatures

**FIGURE 5:**
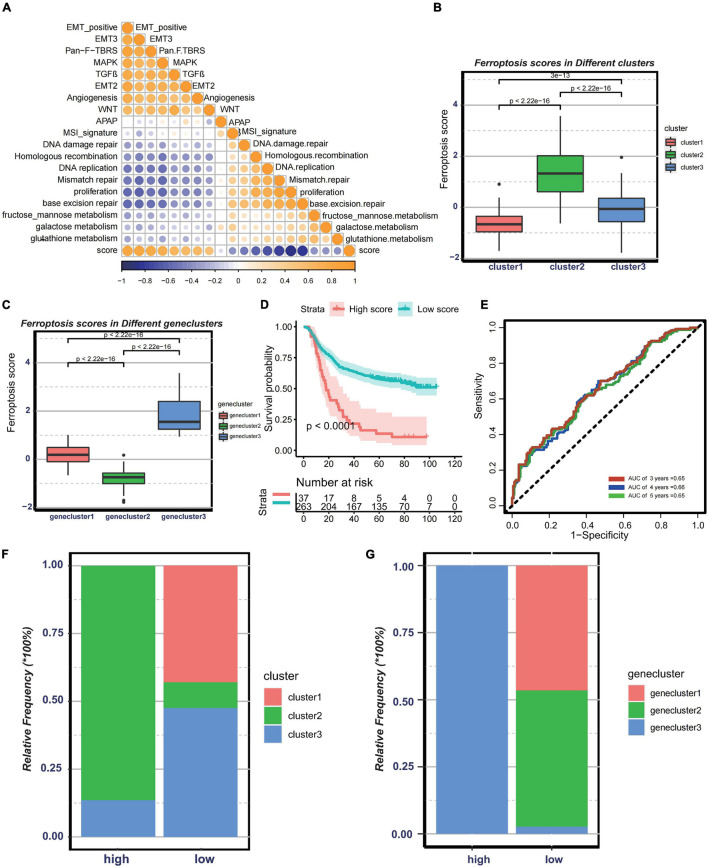
Construction of ferroptosis gene signatures. **(A)** Correlation between known gene signatures related to gastric cancer and the ferroptosis score. **(B)** Differences in the ferroptosis scores among three distinct clusters. **(C)** Differences in the ferroptosis scores among three distinct gene clusters. **(D)** Kaplan–Meier analysis of patients with low or high ferroptosis scores. **(E)** Prognostic value of the ferroptosis score in the Asian Cancer Research Group (ACRG) cohort. **(F)** Relative frequency of clusters in patients with low or high ferroptosis scores in the ACRG cohort. **(G)** Relative frequency of gene clusters in patients with low or high ferroptosis scores in the ACRG cohort.

Since the above-mentioned analyses were based on patient population, it is difficult to accurately identify the phenotypes of individual patients. Considering the complexity of each pattern, we established a DEG-based score, termed ferroptosis score, to quantify the genomic patterns of each GC patient. To illustrate the characteristics of the ferroptosis score, the correlation between the known tumor-related signatures and the ferroptosis score was measured through Spearman’s correlation analysis (Figure 5A). A high ferroptosis score exhibits strong relevance to the stromal activation pathway, whereas a low ferroptosis score is linked to DNA damage repair, metabolism pathways, and antigen processing and presentation. We also discovered that the ferroptosis scores were much higher in cluster 2 (Figure 5B). Besides, patients with high ferroptosis scores tended to be in genecluster 3 (Figure 5C). Therefore, the ferroptosis score model could embody the biological characteristics of each pattern. Then, to determine the clinical value of the ferroptosis score, the patients were divided into two groups—the high ferroptosis score group and the low ferroptosis score group—based on the cutoff values calculated using the “survminer” package. We found that patients in the high ferroptosis score group had unfavorable clinical outcomes compared to those in the low ferroptosis score group (log-rank test: *p* < 0.0001) (Figure 5D). The AUCs of the time-dependent ROC curve of the ferroptosis scores were 0.65, 0.66, and 0.65, respectively, for the 3-, 4-, and 5-year overall survival (Figure 5E). The histogram of the frequency distribution revealed that patients in the low ferroptosis score group mainly comprise patients from cluster 2 or genecluster 3, while those in the high ferroptosis score group mostly come from clusters 1/3 or geneclusters 1/2 with similar clinical outcomes (Figures 5F,G). The results were consistent with the prognostic analyses of clusters or gene clusters, indicating that these three computational methods of classification were rational and had high consistency.

### The Clinical Characteristics Were Associated With the Ferroptosis Score

To validate the stability of the ferroptosis score model (Supplementary Figures 4A–F), the prognostic value of the ferroptosis score signatures was verified in GSE34942 (*n* = 56; log-rank test: *p* < 0.19), GSE57303 (*n* = 70; log-rank test: *p* = 0.061), GSE14549 (*n* = 192; log-rank test: *p* = 0.023), merged GEO GC cohort (*n* = 616; log-rank test: *p* < 0.0001), and the TCGA-STAD cohort (*n* = 350; log-rank test: *p* = 0.0014). The predictive value of the ferroptosis scores for relapse-free survival was also validated in GSE62254 (*n* = 300, *p* < 0.0001). Subsequently, a multivariate Cox regression analysis based on age, TNM stage, and other clinical characteristics as variates was preformed (Supplementary Figure 4G). We found that the ferroptosis score could serve as a robust and independent prognostic indicator to evaluate clinical outcomes in GC [hazard ratio (HR) = 0.3761, 95%CI = 0.2467–0.5733, *p* < 0.001]. This independent prediction effect was also validated in the TCGA-STAD cohort (*HR* = 0.6244, 95%CI = 0.4413–0.8834, *p* = 0.00781) (Supplementary Figure 4H).

**FIGURE 6:**
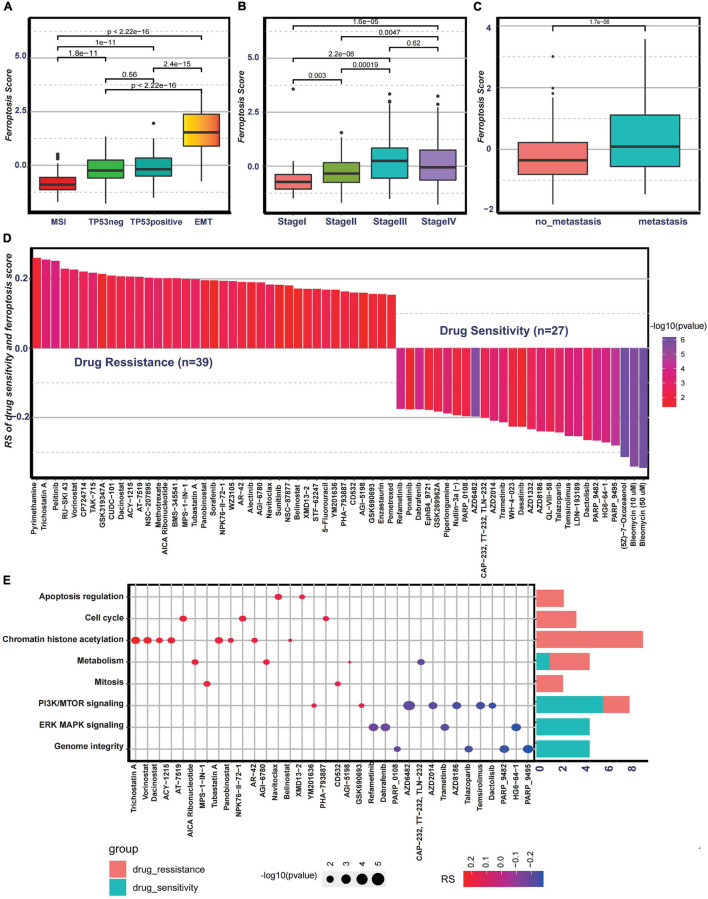
Association between the ferroptosis score and clinical characteristics and the predictive value of the ferroptosis score for drug sensitivity. **(A)** Differences in the ferroptosis scores among the Asian Cancer Research Group (ACRG) molecular subtypes. **(B)** Differences in the ferroptosis scores among different stages in the ACRG cohort. **(C)** Differences in the ferroptosis scores between the metastatic and non-metastatic groups in the ACRG cohort. **(D)** Correlation between the ferroptosis score and drug sensitivity evaluated by Spearman’s analysis. The types of drugs are represented by each *column*. The significance of the correlation between the ferroptosis score and drug sensitivity is represented by the *brightness of the column*. The correlation degree is represented by the *height of the column*. **(E)** Signaling pathways targeted by drugs showing sensitivity (*red*) or resistance (*blue*) to the ferroptosis score. The *size of each point* represents the significance degree of correlation. The *right bar plot* shows the relative frequency of drugs showing sensitivity or resistance to the ferroptosis score in each distinct targeted signaling pathway.

Then, to examine the correlation between the ferroptosis score and ACRG subtypes, the distribution of the ferroptosis scores among the four ACRG subtypes was analyzed. We found that the ferroptosis score was highest in the EMT subtype while lowest in the MSI subtype (Figure 6A). The distribution of the ACRG subtypes between the high ferroptosis score group and the low ferroptosis score group was significantly different (Supplementary Figure 4I). Patients with high ferroptosis scores were mainly of EMT subtypes, while the other subtypes were in the low ferroptosis score group. ACRG subtypes could reflect tumor progression and clinical outcomes, while EMT subtypes were more related to advanced stage tumor and had unfavorable clinical outcomes. We also discovered that the ferroptosis scores varied among the different stages, and tumors in stages III/IV had higher ferroptosis scores (Figure 6B). Moreover, compared to non-metastatic GC patients, patients with metastasis had much higher ferroptosis scores (Figure 6C). Such results revealed that the ferroptosis score could serve as a clinical biomarker to predict tumor progression.

### Potential Predictive Value of Ferroptosis Score for Chemotherapy

To further investigate the potential value of the ferroptosis score for drug response, Spearman’s correlation between the ferroptosis score and drug response in tumor cell lines was analyzed (Figure 6D). The correlation results revealed that 39 drugs exhibited drug resistance associated with the ferroptosis scores, including cell cycle inhibitor AT-7519 (*Rs* = 0.206, *p* = 0.0033), histone deacetylase inhibitor trichostatin A (*Rs* = 0.255, *p* = 0.00043), and adenosine monophosphate analog AICAR (*Rs* = 0.202, *p* = 0.0061), while 27 drugs showed drug sensitivity related to the ferroptosis score, including MAPK signaling inhibitor HG6-64-1 (*Rs* = −0.272, *p* = 0.00017) and phosphatidylinositol-3-kinase (PI3K)/mammalian target of rapamycin (mTOR) signaling inhibitor temsirolimus (*Rs* = −0.0253, *p* = 0.00036) (Figure 6D). The pathways targeted by these drugs are summarized in Figure 6E. We also found that drugs whose sensitivity values were associated with low ferroptosis scores usually targeted cell cycle, mitosis, chromatin histone acetylation, and metabolism, while drugs whose sensitivity scores were associated with high ferroptosis scores usually targeted biological processes, such as MAPK signaling and PI3K/mTOR signaling. In addition, the metabolic subtypes described in the study of Lei et al. (2013) were especially sensitive to 5-fluorouracil. Moreover, it was interesting that 5-fluorouracil exhibited drug resistance correlated with ferroptosis scores, further proving the predictive value of the ferroptosis score on drug response. Overall, these results revealed that the transcriptional features of each pattern were associated with drug response and that the ferroptosis score could serve as a biomarker to predict the drug response of individual patients.

### Predictive Value of Ferroptosis Score for Response to Immunotherapy

**FIGURE 7:**
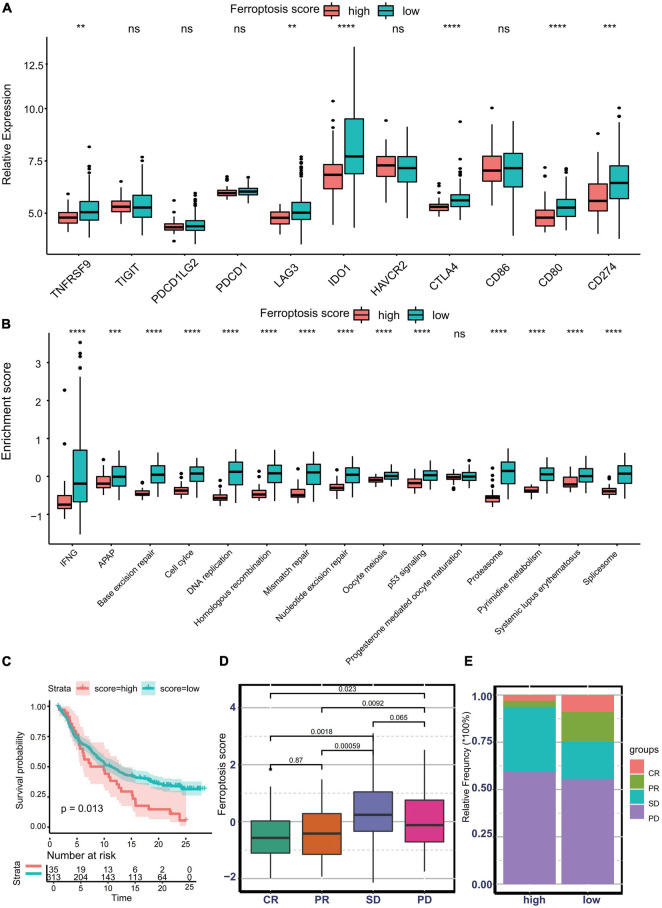
Predictive value of the ferroptosis score for response to immunotherapy. **(A)** Different expression profiles of immune checkpoint-related genes in patients with low or high ferroptosis scores in the Asian Cancer Research Group (ACRG) cohort (**p < 0.01; ***p < 0.001; ****p < 0.0001). *ns*, no significance. **(B)** Different activation status of immunotherapy-positive gene signatures in patients with low or high ferroptosis scores in the ACRG cohort (***p < 0.001; ****p < 0.0001). *ns*, no significance. **(C)** Kaplan–Meier curves showing overall survival in patients with low or high ferroptosis scores in the immunotherapy cohort. **(D)** Distribution of the ferroptosis scores in distinct anti-PD-L1 clinical response groups. **(E)** Relative frequency of each anti-PD-L1 clinical response group in groups with low or high ferroptosis scores.

**FIGURE 8:**
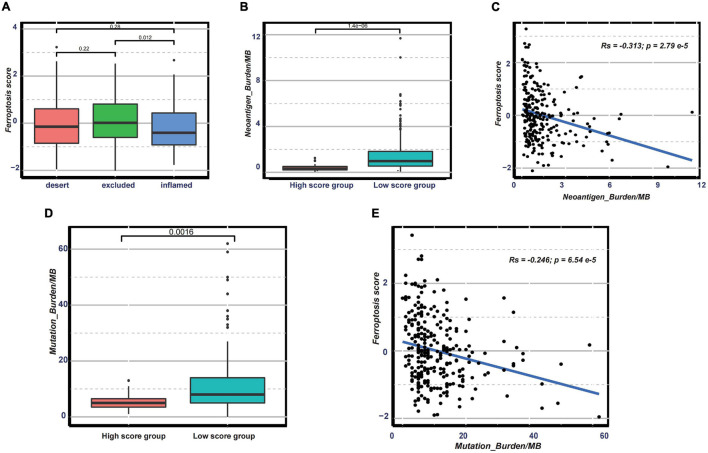
**(A)** Distribution of the ferroptosis scores in distinct immune subtypes in the immunotherapy cohort. **(B–E)** Distribution of the neoantigen burden **(B)** and tumor mutation burden **(D)** in groups with low or high ferroptosis scores and correlations between the ferroptosis score and neoantigen burden **(C)** and between the ferroptosis score and tumor mutation burden **(E)**.

Nowadays, many efforts have been made to identify biomarkers predicting the efficacy of immunotherapy. Based on the current research results, we then analyzed whether the ferroptosis score could serve as a reliable biomarker to predict the efficacy of immunotherapy. In the subsequent analysis, PD-L1, CTLA-4, IDO1, LAG3, HAVCR2, PD-1, PD-L2, CD80, CD86, TIGIT, and TNFRSF9 were selected as targeted immune checkpoints (Zeng et al., 2019). Antigen presentation and processing signaling, base excision repair signaling, cell cycle signature, and other related signaling were chosen as immunotherapy-positive gene signatures (Hu et al., 2021). In the differential expression analysis, PD-L1, CTLA4, IDO1, LAG3, CD80, and TNFRSF9 were significantly upregulated in the low ferroptosis score group (Figure 7A). In addition, the enrichment scores of most immunotherapy-positive signatures were also significantly elevated in the low ferroptosis score group (Figure 7B). These results implied that the ferroptosis score may have a potential role in predicting the efficacy of immunotherapy. In the IMvigor210 cohort, patients with low ferroptosis scores had better clinical outcomes and prolonged overall survival (Figure 7C). The response to PD-L1 blockade was divided into four degrees: complete response (CR), partial response (PR), stable disease (SD), and progressive disease (PD). Patients with CR showed the lowest ferroptosis scores (Figure 7D). The histogram of the frequency distribution also revealed that patients with better clinical responses (CR/PR) were more likely to be in the low ferroptosis score group (Figure 7E). These results suggested that the ferroptosis score could be used to predict clinical response to PD-L1 blockade. Three distinct immune subtypes, termed “immune excluded,” “immune desert,” and “immune inflamed,” were identified in the IMvigor210 cohort. The ferroptosis scores of patients with the immune inflamed subtype were lower than those of the other phenotypes (Figure 8A). Besides, the tumor mutation burden (TMB) and neoantigen burden were significantly higher in the low ferroptosis score group, and both TMB and neoantigen burden showed negative correlations with the ferroptosis score (Figures 8B–E). Accumulating literatures have reported that activation of the immune system and higher levels of TMB and neoantigen burden could benefit immunotherapy (Gibney et al., 2016). Therefore, it may explain why a relatively better clinical response to immunotherapy occurred in patients with low ferroptosis scores. Previous discoveries also revealed that stromal-related pathways that suppressed the functions of immune cells were significantly enriched in the high ferroptosis score group. We speculated that they also play vital roles in resistance to immunotherapy.

## Discussion

Ferroptosis is a form of regulated cell death characterized by the iron-dependent accumulation of lipid hydroperoxides to a lethal level and is regulated by multiple biological processes, such as amino acid, iron and PUFA metabolism, and the biosynthesis of glutathione. Besides, ferroptosis is also involved in the regulation of numerous downstream biological behaviors, such as inflammation, EMT, proliferation, and DNA damage. Therefore, ferroptosis is a complicated biological process and could not be well-delineated by a single regulator. Through Spearman’s correlation analysis, we speculated the presence of a potential regulation relationship among the ferroptosis-related genes, and the interplay among the ferroptosis-related genes also reflected the complexity of ferroptosis. To understand the influence of ferroptosis on tumor cell and TME in depth, integrated biological behaviors of ferroptosis-related genes showing heterogeneous expression levels among GC patients were explored. Identifying the different tumor subtypes determined by ferroptosis could add to our understanding of the biological role of ferroptosis and guide effective therapy.

Based on the expressions of 32 ferroptosis-related genes, we identified three distinct subtypes modified by the different biological behaviors of ferroptosis; each cluster has a distinct molecular characteristic. A significant survival difference among the three clusters was observed, indicating that identifying subtypes with different ferroptosis status has important clinical implications. Pathway enrichment analysis demonstrated that cluster 1 was characterized by the activation of numerous DNA damage repair pathways, cluster 2 exhibited higher enrichment scores for carcinogenic pathways and stromal activation pathways, while cluster 3 showed a pronounced activation of multiple metabolism signatures. Recently, to explore in depth the molecular characteristics of cancer and its correlation with various treatment protocols, several molecular subtypes of GC had been put forward. According to the differential expressions of a series of gene signatures, such as EMT, MSI, cytokine signaling, cell proliferation, DNA methylation, TP53 activity, and gastric tissue, GC could be classified into four molecular subtypes with distinct molecular characteristics and different clinical outcomes. The molecular subtypes included the MSI subtype with hypermutation state, elevated expression of the MSI signature, cytokine signaling, and cell proliferation signaling; the EMT subtype with activation of the EMT signature and abatement of proliferation signatures; the MSS/TP53- subtype; and the MSS/TP53+ subtype (Cristescu et al., 2015). In our study, we also explored the association between consensus molecular subtypes and our own clusters. In the ACRG cohort, the distribution of the EMT subtypes significantly overlapped with cluster 2, while most patients with the MSI subtypes were grouped into cluster 1. Subsequent enrichment analysis also revealed that the biological processes featured by the EMT or the MSI subtypes were also the major characteristics of cluster 2 or cluster 1, respectively. The correlation analysis and pathway enrichment analysis uncovered that cluster 2 is an EMT-like subtype and that cluster 1 is an MSI-like subtype. Moreover, they also implied that ferroptosis may be involved in the regulation of EMT and MSI. A recent study has reported that β-elemene, a new ferroptosis inducer, could lower the expression of the mesenchymal marker, upregulate the expression of the epithelial marker, and suppress migration in colorectal cancer (Chen et al., 2020). The occurrence of ferroptosis is often accompanied by the production and release of ROS, accumulation of which could lead to frameshift mutation, resulting in MSI (Granofszky et al., 2018). The above reports reminded us that ferroptosis may participate in the regulation of EMT and MSI, which is consistent with our discoveries. Tumors in cluster 3 exhibited evident metabolic dysregulation and were more related to the metabolite-like subtypes proposed in the study of Lei et al. (2013). In the preceding differential expression analysis, numerous ferroptosis suppressors, including *AKR1C1*, *AKR1C2*, *AKR1C3*, *MUC1*, *MT1G*, *CA9*, and *PROM2*, were significantly upregulated in cluster 3, implying that cluster 3 is ferroptosis-suppressive. Although a considerable number of studies have paid a lot of attention to the regulatory role of metabolism in ferroptosis, few studies have observed the influence of ferroptosis on metabolism. The existence of a metabolic-like subtype with low activity of ferroptosis implies a mutual regulation between ferroptosis and the metabolism pathways. These results demonstrated that based on the ferroptosis status, tumors could be shaped into three distinct subtypes with unique biological behaviors.

Even if some of the characteristics of the subtypes identified in our study are similar to those of the ACRG genomic subtypes, we also saw several differences in terms of cohort, driver gene, and biological characteristics. We found that tumors with the ACRG MSS/TP53- subtype occupied a substantial proportion in all three clusters; tumors with the MSS/TP53+ subtype were present in clusters 1 and 3. These suggested that the TP53- and TP53+ subtypes were not equal to any subtype in our study. Besides, whereas cluster 1 was characterized by the overactivation of MSI and proliferation signatures, a quite substantial proportion of patients with the MSS/TP53- and MSS/TP53+ subtypes presented in cluster 2. Meanwhile, cluster 2 lacked the activation of immune-activated biological processes, such as antigen processing and presentation, allograft rejection, graft vs. host disease, and RIG-I signaling featured by the MSI subtypes (Supplementary Figures 5A,B), suggesting that cluster 1 and the MSI subtype were probably different. Loss of *CDH1* or *MLH1* was one of the major features in the EMT or the MSI subtype, respectively (Supplementary Figure 5B). Nevertheless, the mRNA expressions of *CDH1* and *MLH1* were not significantly downregulated in cluster 2 (logFC = 0.08, adjusted *p* = 0.89) and cluster 1 (logFC = −0.008, adjusted *p* = 0.99) (Supplementary Figure 5A), further demonstrating that the EMT and MSI subtypes were not equal in cluster 2 and cluster 1, respectively, and that our subtype was unique.

Increasing evidence show that ferroptosis has an indispensable role in inflammation. In our study, each cluster has a distinct landscape of infiltrating immune cells. Unexpectedly, even though cluster 2 had the worse overall survival and relapse-free survival, numerous antitumor immune cells, including central memory CD4 T cells, Th1 cells, natural killer cells, and plasmacytoid dendritic cells, were remarkably enriched in cluster 2. This seemed inconsistent with the dominant perception: abundant infiltration of immune cells is associated with better clinical outcomes. Besides, we also found that a high infiltration of immune cells was accompanied by the activation of the stroma, including the highly expressed TGF-β pathways, angiogenesis, and EMT. Recent studies have found that the activation of the stroma could exclude CD8^+^ T cells from the tumor parenchyma into the fibroblast- and collagen-rich peritumoral stroma (Salmon et al., 2012; Tauriello et al., 2018). Furthermore, elevated TGF-β pathway promoted T cell differentiation toward a subset of regulatory T cells (Tregs) rather than the Th1 effector phenotype and dampened the antigen-presenting function of dendritic cells (Sanjabi et al., 2017). Hence, we speculated that the activation of the stroma was the major cause why cluster 2, with a high infiltration of immune cells, displayed poorer prognosis. Besides, the abundance of activated CD4 T cells and activated B cells increased in clusters 1 and 3, respectively. CD4^+^ T cells relay help signals to CD8^+^ T cells to optimize the magnitude and quality of the response of cytotoxic T lymphocytes (CTLs) within tumor sites (Borst et al., 2018). B cells, the main effector cells of humoral immunity, suppress tumor progression by secreting immunoglobulins, promoting T cell response, and killing cancer cells directly (Tokunaga et al., 2019). Given the above discovery, we considered clusters 1 and 3 as relatively immune-activated compared to cluster 2, and ferroptosis was involved in shaping the TME.

Considering the complexity of individual patterns, a scoring system named ferroptosis score was established to quantify the biological characteristics of each phenotype. A high ferroptosis score exhibits strong relevance to the stromal activation pathway, whereas a low ferroptosis score is linked to DNA damage repair, metabolism pathways, and antigen processing and presentation. Cluster 2, characterized by stromal activation, exhibited a higher ferroptosis score, while cluster 1 presented the lowest ferroptosis score. Similarly, among the ACRG subtypes, the MSI subtype exhibited the lowest ferroptosis score, while the EMT subtype showed the highest ferroptosis score. In the IMvigor210 cohort, the immune-excluded phenotype with activation of the stromal-related pathways also showed a higher ferroptosis score. This suggested that the ferroptosis score is a reliable tool to comprehensively assess the functional role of ferroptosis for individual tumors. Subsequent integrated analysis also revealed that the ferroptosis score could serve as a reliable and robust clinical biomarker to predict the progression and clinical outcomes of GC patients.

Biological features of individual tumors would influence drug response. The correlation between EMT and the drug resistance of cancer cells has been postulated in the early 1990s (Sommers et al., 1992). Multiple genes including TGF-β and CD146 induced EMT through activating the AKT/mTOR pathway, resulting in chemotherapy resistance (Liang et al., 2017; Odero-Marah et al., 2018). Meanwhile, cancer molecular subtypes were proposed to guide effective therapy for patients. For example, in the study of Lei et al. (2013) patients with metabolic subtypes had greater benefits from 5-fluorouracil treatment, while tumors with mesenchymal subtypes were particularly sensitive to PI3K–AKT–mTOR inhibitors *in vitro*. Another study also suggested that temsirolimus significantly inhibits the progression of the EMT subtype renal cell carcinoma in combination with chloroquine (Singla and Bhattacharyya, 2017). Therefore, we also investigated the potential effect of the ferroptosis score on drug response. The ferroptosis score is usually associated with resistance to drugs targeting the cell cycle, chromatin histone acetylation, mitosis, and metabolism and with sensitivity to drugs targeting the ERK/MAPK and PI3K/mTOR pathways. In other words, patients with low ferroptosis scores would benefit from drugs targeting chromatin histone acetylation, mitosis, and metabolism, while patients with higher ferroptosis scores would respond better to drugs targeting the ERK/MAPK and PI3K/mTOR pathways. In the current study, clusters 1 and 3 were associated with low ferroptosis scores, while cluster 2 exhibited high ferroptosis scores. The pathways targeted by drugs whose sensitivity correlated with a low ferroptosis score were usually enriched in clusters 1 and 3; similar results were found for cluster 2. Thus, these indicated that the biological characteristics of each ferroptosis subtype could determine the drug response. Therefore, both the ferroptosis-related subtypes and the ferroptosis score could be regarded as predictors to evaluate the therapeutic effects of chemotherapy. Finally, we also found that the ferroptosis score could predict the response to anti-PD-L1 therapy. Establishment of different ferroptosis-related phenotypes could provide us a new possibility to predict the efficacy of immunotherapy and chemotherapy, promoting personalized treatment for GC patients in the future.

Although previous literatures have reported on the association of ferroptosis-related gene signatures with the TME and prognosis in GC, they have only investigated the roles of several prognostic ferroptosis regulators screened by single Cox regression or LASSO (least absolute shrinkage and selection operator) regression analysis on prognosis and the TME, ignoring the roles of other regulators which are hard to influence prognosis solely. In our study, ferroptosis regulators with MAD < 1 were considered to be constantly expressed among GC patients. Subtyping based on regulators with MAD ≥ 1 allowed us to identify the distinct expression patterns of ferroptosis regulators and to understand the integrated roles of regulators on the TME and prognosis. Therefore, our study could provide a more comprehensive knowledge about ferroptosis compared to other reports.

## Conclusion

In conclusion, based on the expressions of ferroptosis-related genes, three distinct ferroptosis-related patterns of GC were determined in our study. Each pattern has distinct biological characteristics and TME traits. To better depict the features of each pattern, we constructed a scoring system termed ferroptosis score. Through integrated analysis, the ferroptosis score was proven to serve as a reliable biomarker predicting the progression of cancer and the response of individual patients to chemotherapy and immunotherapy. Therefore, our work highlighted the functional role of ferroptosis in tumor cells and the surrounding microenvironment and is conducive to individualized treatment of GC patients.

## Data Availability Statement

The datasets presented in this study can be found in online repositories. The names of the repository/repositories and accession number(s) can be found in the article/Supplementary Material.

## Author Contributions

FW conceived, designed, and supervised the study. SX drafted the manuscript and performed all the data analysis. XL, LY, and XC collected the data. All authors reviewed and approved the final manuscript.

## Conflict of Interest

The authors declare that the research was conducted in the absence of any commercial or financial relationships that could be construed as a potential conflict of interest.

## Publisher’s Note

All claims expressed in this article are solely those of the authors and do not necessarily represent those of their affiliated organizations, or those of the publisher, the editors and the reviewers. Any product that may be evaluated in this article, or claim that may be made by its manufacturer, is not guaranteed or endorsed by the publisher.

## Footnotes

1
https://xenabrowser.net/datapages/

2
http://www.cancerrxgene.org/downloads

## Supplementary Material

Supplementary Figure 1 Ferroptosis-related genes were involved in the development of gastric cancer. **(A)** Expression of ferroptosis-related genes in gastric cancer tissue and normal tissue in TCAG-STAD cohort. **(B)** Principal component analysis of the expression profiles of 32 ferroptosis-related genes to distinguish tumors from normal samples in TCAG-STAD cohort. **(C)** Expression of ferroptosis-related genes in different stages, high grade tumor (stage III/IV) were marked with blue, and low grade tumor (stage I/II) were marked with blue. **(D)** The prognostic analyses for 31 ferroptosis-related genes in the merged gastric cancer cohort using a univariate Cox regression model. **(E)** The correlation among ferroptosis-related genes was shown in heatmap. The asterisks represented the statistical *p-*value (ns represented no significance; ^∗^*p* < 0.05; ^∗∗^*p* < 0.01; ^∗∗∗^*p* < 0.001).

Supplementary Figure 2 Genetic alteration of 32 ferroptosis-related genes in gastric cancer. **(A)** The copy number variation and relative frequency for 32 ferroptosis-related genes in TCGA-STAD cohort. **(B)** The mutation profile of ferroptosis-related genes in TCGA-STAD cohort.

Supplementary Figure 3 Unsupervised clustering of 32 ferroptosis-related genes in ACRG cohort. **(A–D)** Consensus clustering matrix of ACRG cohort for *k* = 2–5. **(E)** Kaplan-Meier analysis of patients among three different ferroptosis-related clusters in ACRG cohort. **(F)** Relapse-free survival analysis of patients among three different ferroptosis-related clusters in ACRG cohort. **(G)** 933 phenotype-related genes were shown in Venn diagrams.

Supplementary Figure 4 Prognostic value of ferroptosis score in gastric cancer cohort. **(A–E)** Kaplan-Meier curves show overall survival in patients with low or high ferroptosis score in GSE34942, GSE57303, GSE14549, merged GEO GC cohort and TCGA-STAD cohort, respectively. **(F)** Relapse-free survival analysis of patients with low or high ferroptosis score in ACRG cohort. **(G,H)** Multivariate Cox regression model analysis including ferroptosis score, age, gender, TNM status in the ACRG cohort **(G)** and TCGA-STAD cohort **(H)**. **(I)** The relative frequency of ACRG molecular subtypes in groups with low or high ferroptosis score.

Supplementary Figure 5 Different activation status of immune-related pathways and driver genes between ACRG subtype and clusters of our study. **(A)** Difference in activated status of immune-related pathways and driver genes among clusters. **(B)** Difference in activated status of immune-related pathways and driver genes among ACRG subtypes.

## Abbreviations

GC: gastric cancer
GEO: Gene Expression Omnibus
TCGA: The Cancer Genome Atlas
MSI: microsatellite instability
EMT: epithelial–mesenchymal transition
GSH: glutathione
ROS: reactive oxygen species
NADPH: nicotinamide adenine dinucleotide phosphate
TME: tumor microenvironment
DAMPs: damage-associated molecular patterns
MAD: median absolute deviation
NMF: non-negative matrix factorization
GSVA: gene set variation analysis
DEGs: differentially expressed genes
ssGSEA: single-sample gene set enrichment analysis
TGF- β: transforming growth factor beta
Pan-F-TBRS: pan-fibroblast TGF- β response signature
ACRG: Asian Cancer Research Group
GDSC: Genomics of Drug Sensitivity in Cancer database
ROC: receiver operating characteristic
AUC: area under the ROC curve
PCA: principal component analysis
CNV: copy number variation
MDSC: myeloid-derived suppressor cell
TLR: Toll-like receptor
NLR: NOD-like receptor
TMB: tumor mutation burden.
