# Supplementary figures and images for: Expression of Ferroptosis-Related Genes Shapes Tumor Microenvironment and Pharmacological Profile in Gastric Cancer
Source: Front Cell Dev Biol. 2021 Oct 1;9:694003. doi: 10.3389/fcell.2021.694003 (PMC8517126; doi:10.3389/fcell.2021.694003)

Figure S1

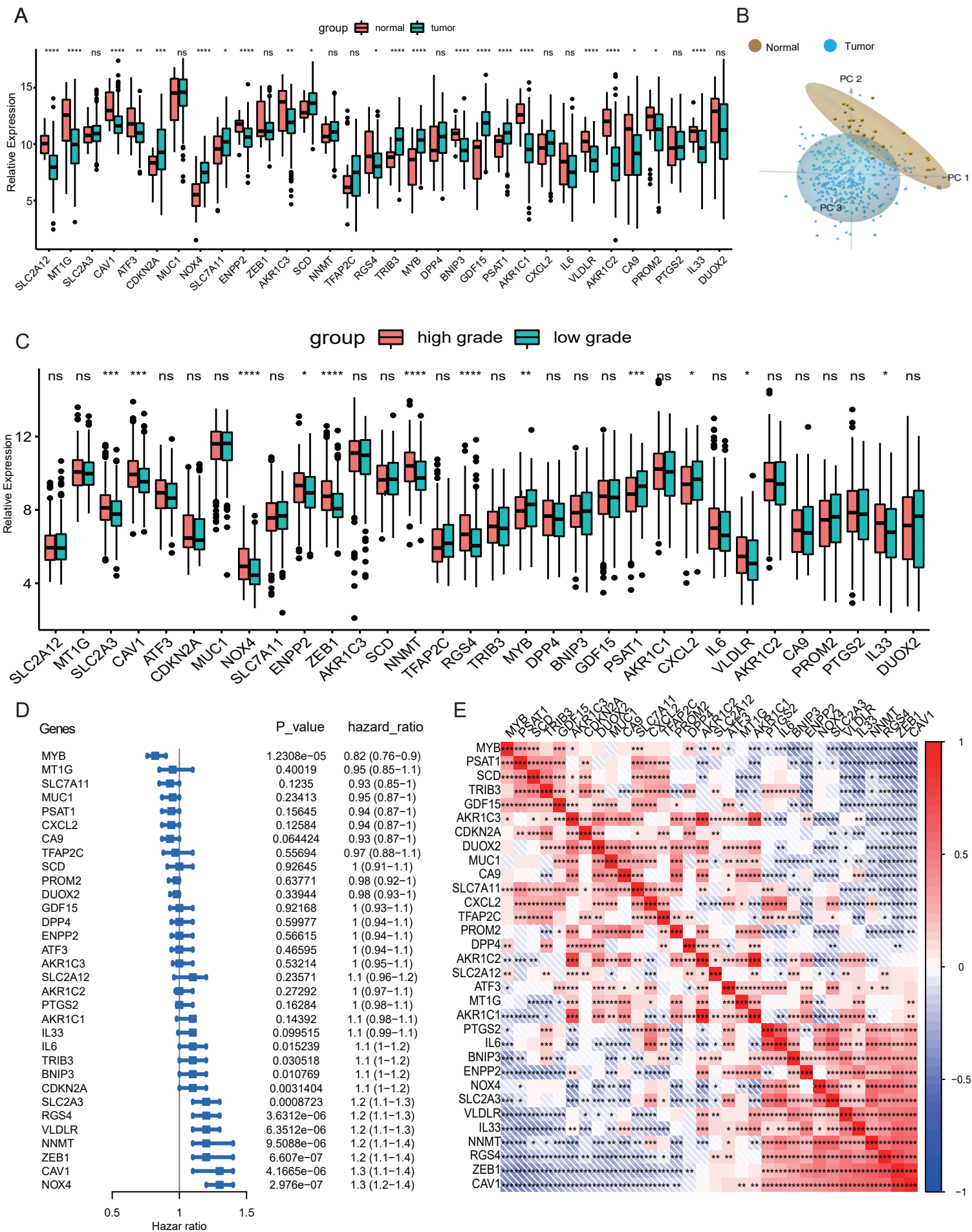

Supplement: Supplementary Figure 1 — Ferroptosis-related genes were involved in the development of gastric cancer. (A) Expression of ferroptosis-related genes in gastric cancer tissue and normal tissue in TCAG-STAD cohort. (B) Principal component analysis of the expression profiles of 32 ferroptosis-related genes to distinguish tumors from normal samples in TCAG-STAD cohort. (C) Expression of ferroptosis-related genes in different stages, high grade tumor (stage III/IV) were marked with blue, and low grade tumor (stage I/II) were marked with blue. (D) The prognostic analyses for 31 ferroptosis-related genes in the merged gastric cancer cohort using a univariate Cox regression model. (E) The correlation among ferroptosis-related genes was shown in heatmap. The asterisks represented the statistical p-value (ns represented no significance; ∗p < 0.05; ∗∗p < 0.01; ∗∗∗p < 0.001). [file Data_Sheet_1.PDF]

Figure S2

A

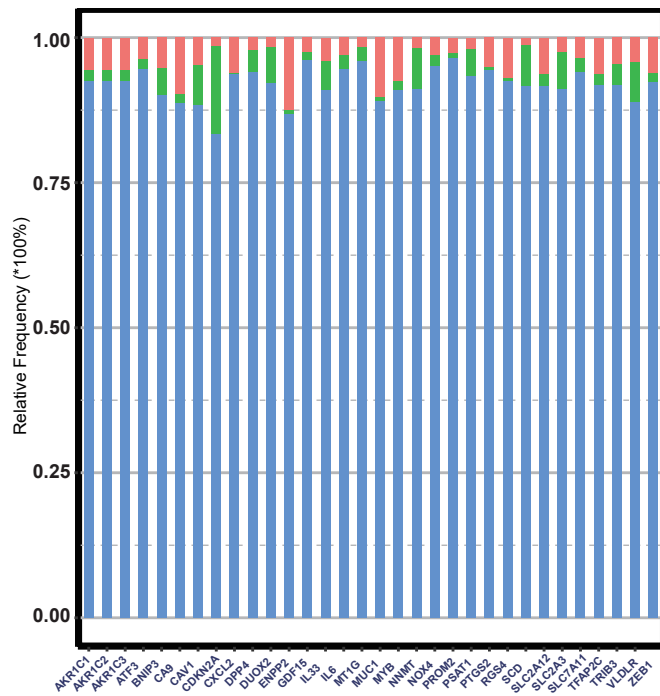

B

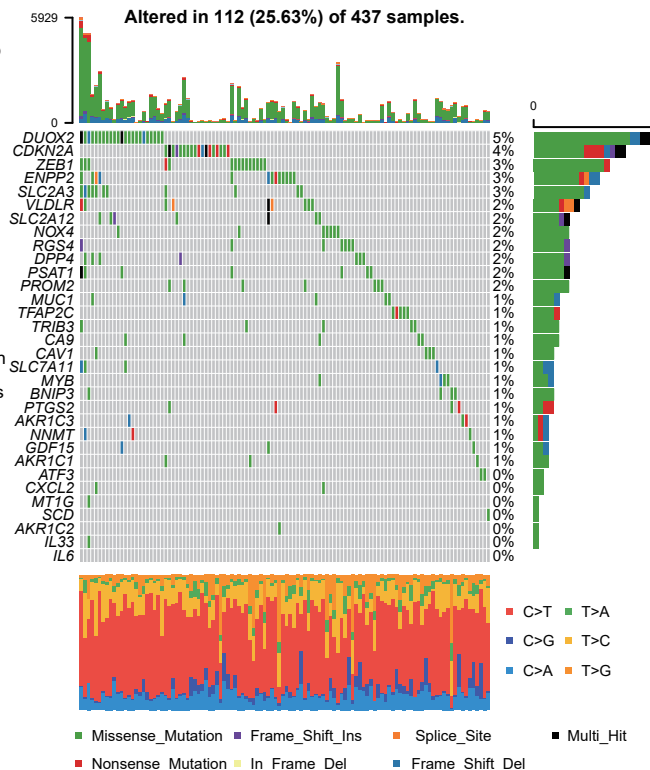

Supplement: Supplementary Figure 2 — Genetic alteration of 32 ferroptosis-related genes in gastric cancer. (A) The copy number variation and relative frequency for 32 ferroptosis-related genes in TCGA-STAD cohort. (B) The mutation profile of ferroptosis-related genes in TCGA-STAD cohort. [file Data_Sheet_2.PDF]

Figure S3

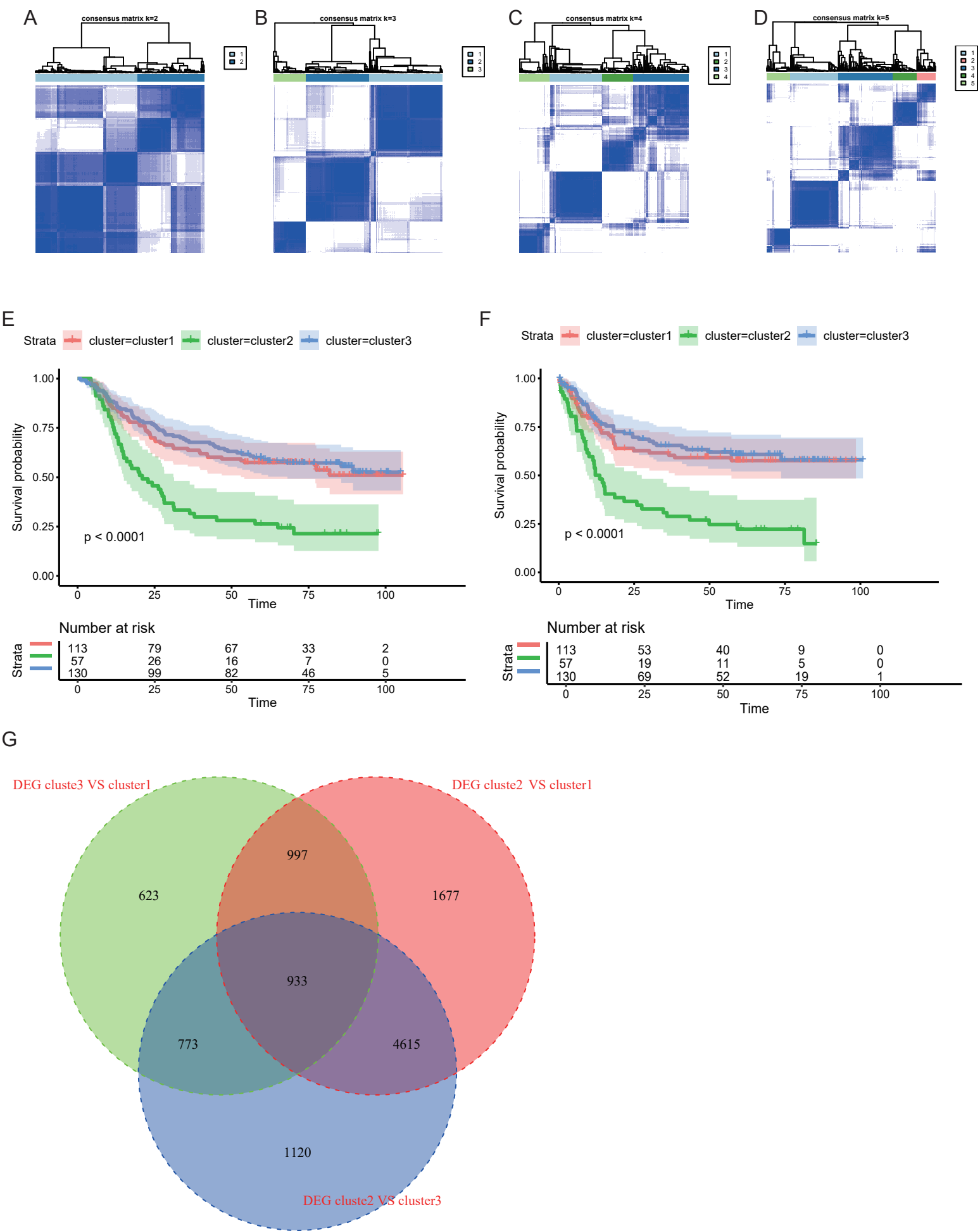

Supplement: Supplementary Figure 3 — Unsupervised clustering of 32 ferroptosis-related genes in ACRG cohort. (A–D) Consensus clustering matrix of ACRG cohort for k = 2–5. (E) Kaplan-Meier analysis of patients among three different ferroptosis-related clusters in ACRG cohort. (F) Relapse-free survival analysis of patients among three different ferroptosis-related clusters in ACRG cohort. (G) 933 phenotype-related genes were shown in Venn diagrams. [file Data_Sheet_3.PDF]

Figure S4

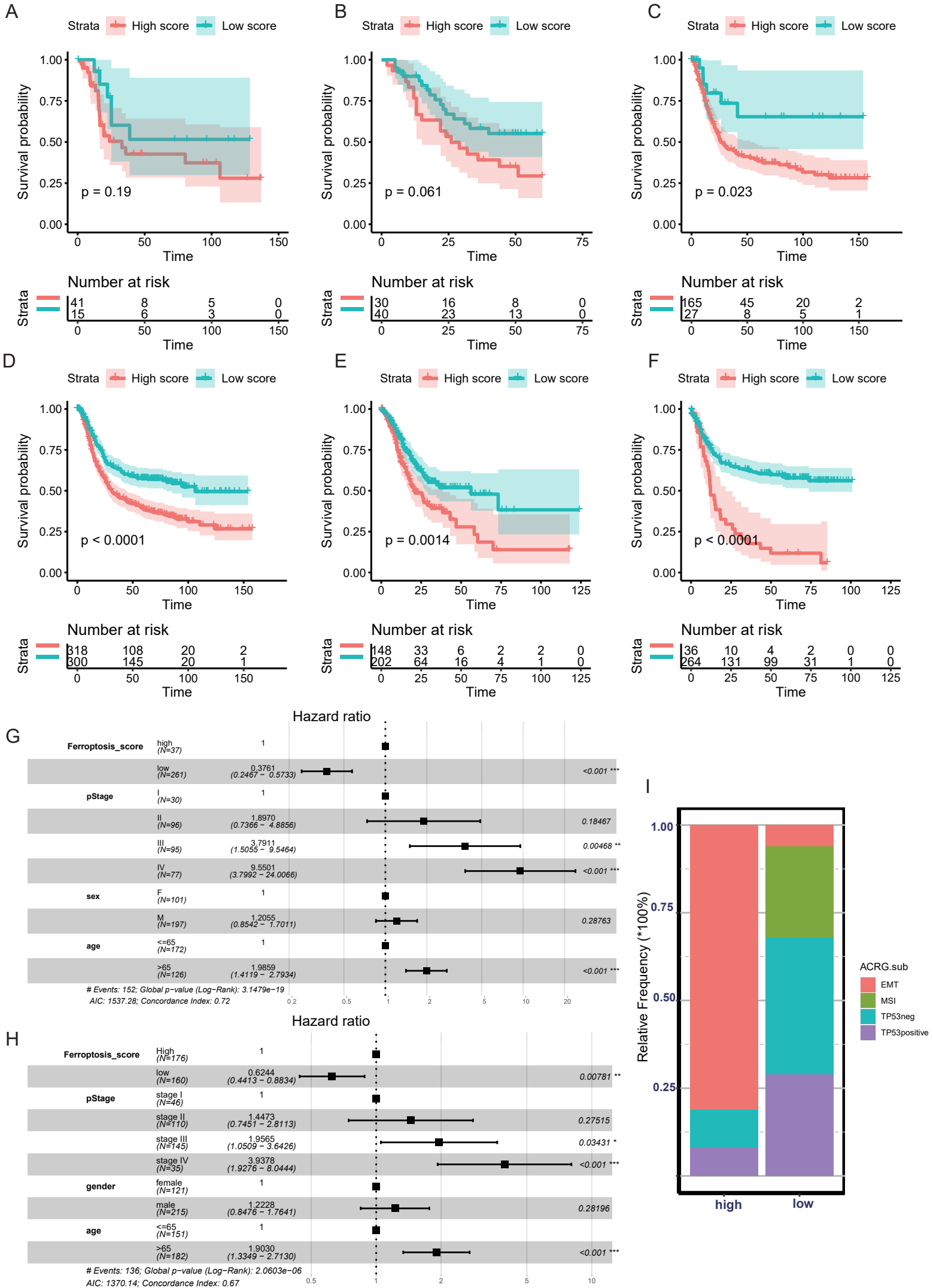

Supplement: Supplementary Figure 4 — Prognostic value of ferroptosis score in gastric cancer cohort. (A–E) Kaplan-Meier curves show overall survival in patients with low or high ferroptosis score in GSE34942, GSE57303, GSE14549, merged GEO GC cohort and TCGA-STAD cohort, respectively. (F) Relapse-free survival analysis of patients with low or high ferroptosis score in ACRG cohort. (G,H) Multivariate Cox regression model analysis including ferroptosis score, age, gender, TNM status in the ACRG cohort (G) and TCGA-STAD cohort (H). (I) The relative frequency of ACRG molecular subtypes in groups with low or high ferroptosis score. [file Data_Sheet_4.PDF]

Figure S5

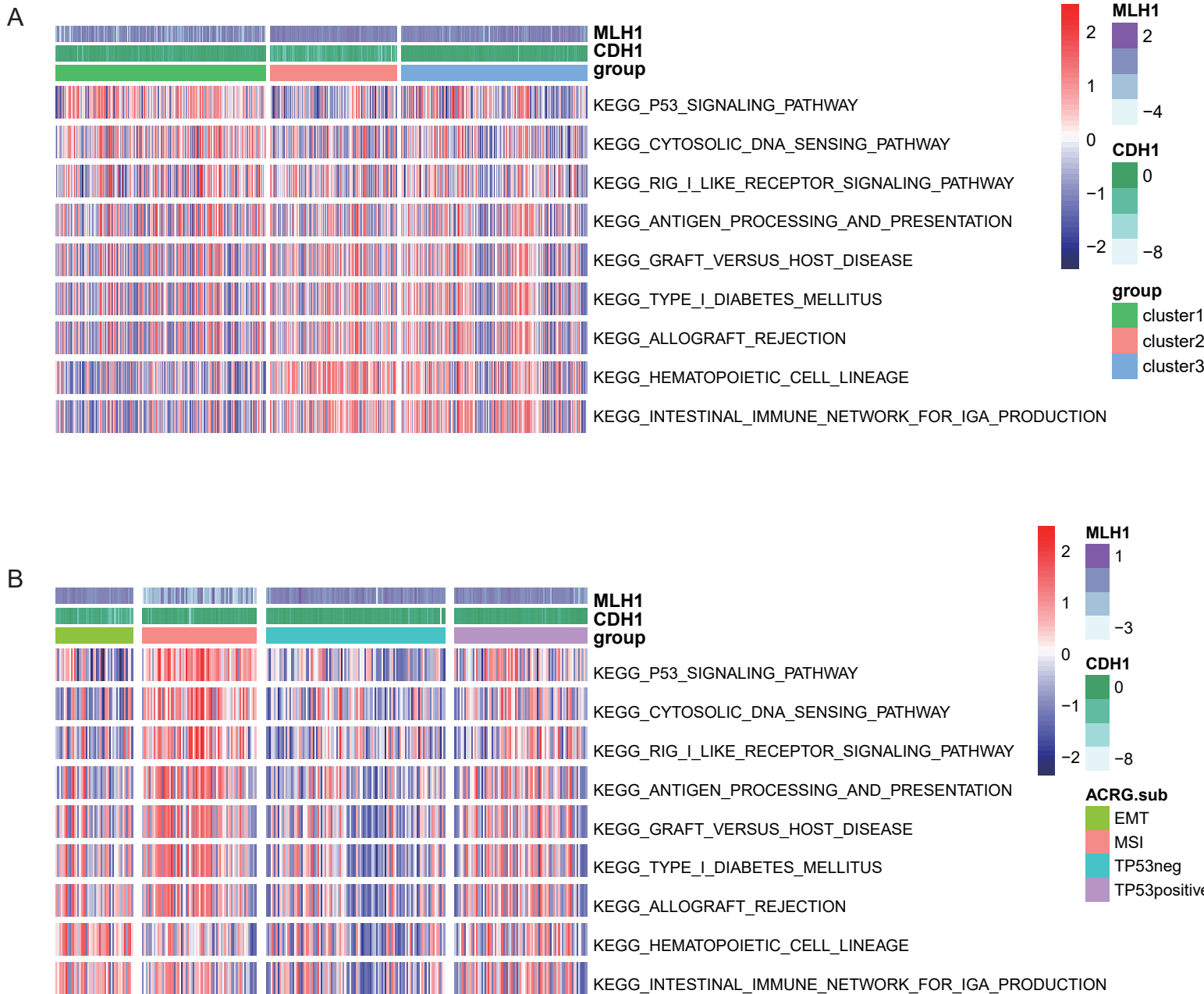

Supplement: Supplementary Figure 5 — Different activation status of immune-related pathways and driver genes between ACRG subtype and clusters of our study. (A) Difference in activated status of immune-related pathways and driver genes among clusters. (B) Difference in activated status of immune-related pathways and driver genes among ACRG subtypes. [file Data_Sheet_5.PDF]
